# Development of a Loop-Mediated Isothermal Amplification (LAMP) for the screening of *Candida auris*

**DOI:** 10.1371/journal.pone.0348003

**Published:** 2026-04-24

**Authors:** Woong Sik Jang, Young Lan Choe, Soo Young Yoon, Chae Seung Lim, Min-Chul Cho

**Affiliations:** 1 Emergency Medicine, College of Medicine, Korea University Guro Hospital, Seoul, Republic of Korea; 2 Department of Laboratory Medicine, Korea University Guro Hospital, Korea University College of Medicine, Seoul, Republic of Korea; Kwame Nkrumah University of Science and Technology, GHANA

## Abstract

**Background:**

*Candida auris* is an emerging multidrug-resistant yeast associated with invasive infections, healthcare-associated outbreaks, and high mortality, and is often misidentified by conventional diagnostic methods. Rapid, accurate, and scalable screening tools are essential for effective infection control, particularly in high-risk settings.

**Materials and methods:**

We developed a multiplex loop-mediated isothermal amplification (LAMP) assay that combines a broad-range *Candida* Pan target with a *C. auris*–specific target in a single isothermal reaction. Assay conditions were optimized for primer ratio and temperature, and analytical sensitivity was evaluated using serial dilutions of culture-derived *C. albicans* and *C. auris* DNA, as well as contrived specimens consisting of urine, swab, and whole-blood matrices. Clinical performance was assessed using 35 *Candida*-positive clinical specimens (blood, urine, ear swabs) and 94 non-infectious controls. Results were compared with *Candida* Pan qPCR and *C. auris* qPCR. Cross-reactivity was tested against common bacterial isolates.

**Results:**

Under optimized conditions (1:1 primer ratio, 64 °C), the assay allowed species-level discrimination, with *C. auris* positive for both Pan and *auris* channels and *C. albicans* positive only for the Pan channel. The *C. auris*-specific LAMP probe detected approximately 10²–10³ cells/mL in culture-derived and contrived specimens, showing a 1–2 log improvement over *C. auris* qPCR (10⁴–10⁵ cells/mL), while the Pan LAMP channel detected *C. auris* at around 10⁵ cells/mL. In clinical specimens, Pan LAMP detected *Candida* spp. in 34/35 cases (97.14%) versus 32/35 (91.14%) for Pan qPCR. All *C. auris*–positive specimens (9/9) were detected by the multiplex LAMP assay, compared with 6/9 (66.7%) by Pan qPCR. All 94 non-infectious controls and all bacterial isolates tested negative, indicating 100% clinical specificity and absence of cross-reactivity.

**Conclusion:**

The multiplex *Candida* Pan/*auris* LAMP assay provides a rapid, highly sensitive, and specific alternative to qPCR for *C. auris* screening, while preserving broad *Candida* detection in a single isothermal reaction. Its improved analytical and clinical sensitivity suggests strong potential for use in active surveillance and infection-control programs, particularly in settings where timely identification and containment of *C. auris* are critical.

## Introduction

*Candida auris* is a multidrug-resistant opportunistic yeast that was first described in 2009 after isolation from the external ear canal of an inpatient in Japan and has since emerged globally as a major healthcare-associated pathogen [1]*.* This pathogen has since spread globally and become recognized as a critical antimicrobial resistance threat due to its unique epidemiological characteristics that distinguish it from other *Candida* species [2]. Misidentification by conventional diagnostic platforms and the organism’s exceptional capacity for human-to-human transmission have complicated clinical management and infection-control responses, prompting widespread adoption of active surveillance strategies in healthcare facilities [3]. The pathogen demonstrates remarkable environmental persistence, surviving on hospital surfaces for weeks to months, which facilitates prolonged nosocomial outbreaks affecting vulnerable patient populations [2,4].

*C. auris* infections predominantly affect immunocompromised patients in intensive care units, with intrinsic resistance to multiple antifungal classes—approximately 90% of isolates are resistant to fluconazole, 30% resistant to amphotericin B, and emerging echinocandin resistance were reported in some regions [5,6]. The clinical significance of rapid and accurate *C. auris* detection becomes paramount given mortality rates ranging from 30–72% in patients with invasive infections, making timely identification absolutely critical for appropriate antifungal therapy selection, patient management, and implementation of infection prevention measures [3,6]

Recent reviews have highlighted that screening, identification, and susceptibility testing of *C. auris* remain challenging in routine clinical laboratories, particularly because of frequent misidentification and the need for rapid infection-control responses [7]. Traditional phenotypic and biochemical identification systems may misidentify *C. auris*, whereas MALDI-TOF MS provides improved accuracy when updated reference databases are available. However, MALDI-TOF MS and PCR-based assays may still be limited in some settings by instrument cost, workflow complexity, or infrastructure requirements. These limitations underscore the need for rapid, cost-effective, and accurate screening methods for timely infection control and patient management [7,8]. These limitations collectively emphasize the urgent need for rapid, cost-effective, and accurate screening methods that can provide timely results for effective infection control and patient management.

Loop-mediated isothermal amplification (LAMP) may offers a promising molecular diagnostic platform that addresses many of the limitations associated with current *C. auris* detection methods. LAMP is an isothermal nucleic acid amplification method performed at a single temperature, typically 60–65 °C, using four to six primers that recognize multiple regions of the target sequence. Because of its speed, simplicity, and high analytical specificity, it has been widely investigated as a practical molecular platform for pathogen detection in laboratory and near-patient settings [9,10]. Compared with conventional PCR-based approaches, LAMP can tolerate some inhibitory substances present in clinical materials, and direct detection from minimally processed or crude samples has been demonstrated in several assay formats [11,12]. The amplification products can be detected through multiple methods including turbidimetry, fluorescence, colorimetric changes, or even visual inspection by the naked eye, eliminating the need for sophisticated detection equipment [13,14].

In this study, we developed a LAMP-based screening assay for direct detection of *C. auris* and applied it across diverse clinical specimen types—including blood, urine, and surveillance swabs. The assay showed no cross-reactivity with other clinically important *Candida* species and other bacteria that are commonly associated with co-infections. By leveraging LAMP’s abbreviated reaction time, streamlined workflow, and compatibility with simpler instrumentation, the assay is positioned to support timely cohorting and contact precautions, enhancing infection-prevention and hospital-management workflows where rapid, scalable identification of colonized or infected patients is essential.

## Materials and methods

### Clinical specimens, clinical isolates, and microbial strains

Clinical specimens were collected at Korea University Guro Hospital between 01/01/2019 and 27/07/2025, including 35 *Candida*-positive specimens (whole blood, n = 9; urine, n = 13; ear swabs, n = 13) and 94 negative control specimens, for a total of 129 specimens. Species identification was carried out using the VITEK 2 COMPACT system (bioMérieux, Durham, NC, USA) with the VITEK®2 YST ID card. *Candida*-positive isolates included *C. albicans* (n = 6), *C. glabrata* (n = 6), *C. parapsilosis* (n = 5), *C. tropicalis* (n = 7), *C. krusei* (n = 1), *C. utilis* (n = 1), and *C. auris* (n = 9). All identification test results were derived from routine clinical microbiology identification tests performed for the medical care of patients who visited Korea University Guro Hospital. These data were obtained retrospectively by reviewing the Korea University Guro Hospital electronic medical records (EMR) during the period from 01/09/2025–26/09/2025.

Reference *Candida* strains used in this study were *C. albicans* (CCARM 14029) and *C. auris* (KCTC 17850), which were obtained from the Culture Collection of Antimicrobial-Resistant Microbes (CCARM; Seoul, Korea) and the Korean Collection for Type Cultures (KCTC; Daejeon, Korea), respectively. For cross-reactivity testing, we used bacterial isolates recovered from clinical specimens of patients processed at the clinical microbiology laboratory, Department of Laboratory Medicine, Korea University Guro Hospital, including *Escherichia coli*, *Enterococcus faecium*, *Enterococcus faecalis*, *Staphylococcus aureus*, *Staphylococcus epidermidis*, *Streptococcus pneumoniae*, and *Pseudomonas aeruginosa*. For specificity and cross-reactivity testing, bacterial and fungal organisms were selected based on their clinical relevance and frequency in routine diagnostic microbiology. In particular, the panel was designed to include microorganisms commonly isolated and identified from clinical specimens in our clinical laboratory setting. Among fungal pathogens, *Candida* species are the most commonly recovered yeasts from clinical specimens, and *C. albicans* was included as a representative species because of its high prevalence and clinical importance. This retrospective study used bacterial isolates previously obtained from patients’ clinical specimens. It was conducted in accordance with the Declaration of Helsinki and approved by the Institutional Review Board of Korea University Guro Hospital (2025GR0392) with an exemption from review and a waiver of informed consent.

### DNA Extraction from *Candida* Strains and clinical specimens

Genomic DNA was extracted from *Candida* reference strains and clinical specimens using the QIAamp UCP Pathogen Mini Kit (Qiagen, Hilden, Germany) according to the manufacturer’s protocol. Candida strains were cultured overnight in yeast peptone dextrose (YPD) broth (Difco BD, Milan, Italy) at 37 °C with agitation at 180 rpm, and cell densities (~1 × 10^7^ cells/mL) were estimated by phase-contrast microscopy (40×) using a counting grid. For clinical specimens, ear swabs and urine samples were centrifuged to obtain cell pellets, while blood samples were pretreated with Red Blood Cell Lysis Buffer (1:4, v/v; Bio Basic, Toronto, ON, Canada) for 10 min to remove erythrocytes and subsequently centrifuged at 4,000 × g for 10 min. The resulting pellets were processed with the QIAamp kit, which involves enzymatic digestion and protease treatment, DNA binding to a silica membrane, sequential washing, and elution. DNA concentration and purity were assessed spectrophotometrically, and extracts were stored at –20 °C until further use.

### Primer design

The *Candida* pan LAMP primer set was adapted from a previously published assay targeting the conserved ITS1–5.8S–ITS2 regions specific to the *Candida* genus by aligning sequences from six *Candida* species (*C. albicans* MT640022.1:70–499, *C. glabrata* MT548912.1:350–885, *C. krusei* MZ507554.1:50–538, *C. tropicalis* LC639851.1:50–601, *C. parapsilosis* LC641867.1:130–786, and *C. auris* OL455790.1:1–300) with minor modifications [15]. Briefly, the loop forward primer (FLP) was repositioned, and the fluorescence probe was redesigned based on the FLP sequence to support multiplex integration. For *C. auris*, primers were designed within conserved regions of the 28S rRNA gene (NG055302.1:16–237), with primer specificity confirmed through sequence alignment among *Candida* species prior to LAMP testing. Each LAMP set consisted of two outer primers (F3 and B3), two inner primers (FIP and BIP), and two loop primers (LF and LB), designed using Primer Explorer software (Version 4; Eiken Chemical Co., Tokyo, Japan). For the multiplex LAMP assay, *Candida* species was detected using strand-displaceable fluorophore–quencher probe pairs consisting of a Pan FLP probe 1 labeled with FAM at the 5′ end and a complementary quencher probe 1 labeled with BHQ1 at the 3′ end. In contrast, *C. auris* was detected using hybridization-based TaqMan-style probes (HyTaq), in which the FLP sequence was dual-labeled with Cy5 at the 5′ end and BHQ2 at the 3′ end. All primers and probes were synthesized by Macrogen, Inc. (Seoul, South Korea) and are listed in Table 1.

**Table 1:** Primer sets used for multiplex *Candida* Pan/*auris* LAMP and qPCR assays.

Target	Name	Sequence (5`-3`)	Length (mer)
*Candida* Pan (18s rRNA)	CPAN F3	CGA TAC GTA ATA TGA ATT GCA GAT	24
	CPAN B3	AGA CCT AAG CCA TTG TCA A	19
	CPAN FIP	AAC GAC GCT CAA ACA GGC ATC GTG AAT CAT CGA ATC TTT GAA C	43
	CPAN BIP	CTG GGT TTG GTG TTG AGC AAA TCC CGC CTT ACC ACT AC	38
	CPAN FLP	CAG AGG GCG CAA TGT GC	17
	CPAN BLP	ACG ACT TGG GTT TGC TTG AAA	21
	CPAN FLP probe 1	[FAM] – CGG GCC CGT ACA AAG GGA ACA CCC ACA CTC CGC AGA GGG CGC AAT GTG C	53
*Candida auris* (28s rRNA)	Cauris F3	GGA TTG CCT CAG TAA CGG C	19
	Cauris B3	GCT GCA TTC CCA AAC AAC TC	20
	Cauris FIP	TGG TGG CCA CCT CCA GAC TAC GAG TGA AGC GGC AAG AGC	39
	Cauris BIP	TAG CAG CAG GCA AGT CCT TTG GGC CAC AGG AAG CAC TAG C	40
	Cauris BLP	AAC AAG GCG CCA GCG A	16
	Cauris FLP	CGG AGC GAT TCC AAA GTT GA	20
	Cauris FLP probe 2	[Cy5] – CGG AGC GAT TCC AAA GTT GA-BHQ2	55
Quencher probe 1		GAG TGT GGG TGT TCC CTT TGT ACG GGC CCG -BHQ1	30
*Candida* Pan PCR [16]	Candida Pan F	CCT GTT TGA GCG TCR TTT	18
	Candida Pan R	TCC TCC GCT TAT TGA TAT	18
*Candida auris* PCR [17]	Cauris F	CGT GAT GTC TTC TCA CCA ATC T	22
	Cauris R	TAC CTG ATT TGA GGC GAC AAC	21

LAMP: Loop-mediated isothermal amplification; F3: forward primer; B3: backward primer; FIP: forward inner primer; BIP: backward inner primer; LF: loop forward primer; LB: loop backward primer; LBP: loop backward probe

### The *Candida* Pan/*auris* LAMP assay

The *Candida* Pan/*auris* LAMP assay was carried out using the Mmiso DNA amplification kit (Mmonitor, Daegu, South Korea). For multiplex reactions, each 25 μL mixture contained 12.5 μL of 2 × reaction buffer, 1.25 μL of *Candida* Pan primer mix (20×), 1.25 μL of *C. auris* primer mix (20×), 1.25 μL of a 9 μM quencher solution targeting the Pan probe, and 2 μL of DNA template. The *Candida* Pan primer mix (20×) consisted of two outer primers (F3 and B3, 4 µM each), two inner primers (FIP and BIP, 32 µM each), a loop forward primer (FLP, 10 µM), a loop backward primer (BLP, 4 µM), and an FLP-linked FAM probe (6 µM). The *C. auris* primer mix contained two outer primers (F3 and B3, 4 µM each), two inner primers (FIP and BIP, 32 µM each), loop primers (FLP and BLP, 10 µM each), and an FLP-linked Cy5 probe (10 µM). Amplification was carried out at 64 °C for 40 min using a CFX96 Touch Real-Time PCR Detection System (Bio-Rad Laboratories, Hercules, CA, USA).

### Real-Time PCR

The performance of the *Candida* Pan/IC LAMP assay was compared with real-time PCR assays targeting *Candida* pan [16] and *Candida auris* [17] (Table 1). Reactions were run using the iQ Multiplex Powermix (Bio-Rad Laboratories, California, USA) on a CFX96 Touch Real-Time PCR Detection System (Bio-Rad Laboratories) with the following cycling program: 50 °C for 2 min, 95 °C for 10 min, and 39 cycles of 95 °C for 15 s and 60 °C for 1 min with fluorescence acquisition.

### Analytical detection performance

The analytical detection performance of the *Candida* Pan/*auris* LAMP assay was assessed using serially diluted. The clinically relevant detection performance and contrived specimens. Genomic DNA extracted from 1.0 × 10⁷ cells of *C. albicans* and *C. auris* was quantified using a NanoDrop spectrophotometer (Thermo Fisher Scientific, USA), and both preparations yielded concentrations of 8 ng/µL. Defined DNA inputs ranging from 16 ng to 0.0016 pg per reaction were generated by 10-fold serial dilution using distilled water (DW). To evaluate analytical performance in contrived specimens, blood, ear swab, and urine matrices were artificially inoculated with defined concentrations of *C. albicans* or *C. auris* (10⁷ to 10^0^ cells/mL), followed by nucleic acid extraction. Detection ranges obtained using the multiplex Pan/*auris* LAMP assay were compared with those obtained using conventional PCR assays for *Candida* Pan and *C. auris* (Table 1). All experiments were performed in triplicate.

### Statistical analysis

Organism identification results obtained by the routine clinical reference methods used in our laboratory, including VITEK 2 and MALDI-TOF MS, were regarded as the reference standard. Based on this reference, the performance of the *Candida* Pan/*auris* LAMP assay was evaluated using analytical and clinical performance measures, including the limit of detection, clinical sensitivity, and clinical specificity. Clinical sensitivity was defined as the proportion of reference-positive specimens correctly identified by the assay, and clinical specificity was defined as the proportion of reference-negative specimens correctly identified by the assay. In this context, the true positive rate and true negative rate correspond to clinical sensitivity and clinical specificity, respectively.

## Results

### Optimization of the *Candida* PAN/*auris* LAMP primer sets

**Fig 1:**
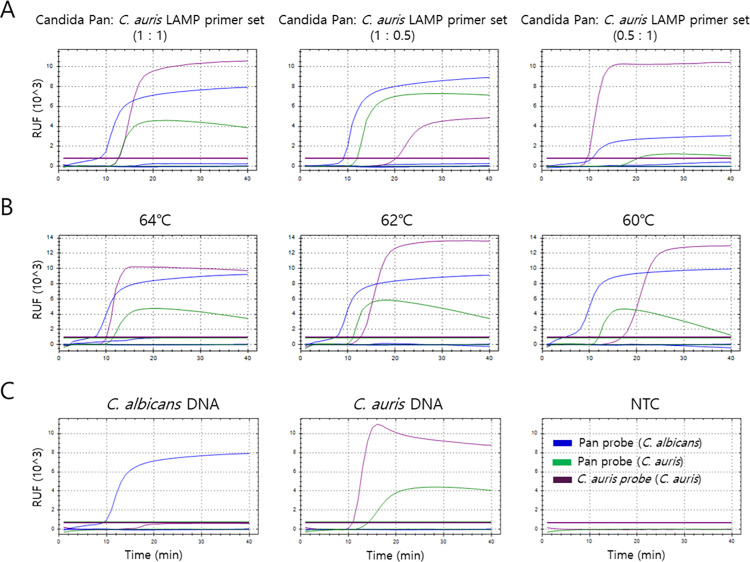
Optimization of the *Candida* Pan/*auris* LAMP assay.

The primer composition and reaction temperature of the *Candida* Pan/*auris* LAMP assay were optimized prior to analytical validation (Fig. 1). When three primer mixing ratios of the *Candida* Pan and *C. auris*–specific primer sets (1:1, 1:0.5, and 0.5:1) were compared, the 1:1 ratio yielded the most consistent and balanced amplification signals for both targets, the broad-range *Candida* Pan target and the *C. auris*–specific target (Fig. 1A). Temperature screening further demonstrated that 64 °C produced the most stable and reproducible amplification profiles for both the *Candida* Pan and *C. auris*–specific targets, compared with 60 °C and 62 °C (Fig. 1B). Under these optimized conditions, *C. albicans* generated amplification only with the *Candida* Pan probe, whereas *C. auris* generated amplification with both the Pan and *auris*-specific probes, confirming species-level differentiation within the multiplex reaction (Fig. 1C).

In the amplification curves, the Pan probe fluorescence (FAM) was displayed in blue for *C. albicans* and in green for *C. auris* to visually distinguish the two species, while the *C. auris*–specific probe signal (Cy5) was displayed in magenta. (Note: Colors are assigned for visual differentiation and do not represent actual emission wavelengths.) (A) Primer composition optimization of the Candida Pan/auris LAMP assay. Three ratios of the Candida pan and *C. auris*–specific primer sets (1:1, 1:0.5, and 0.5:1) were evaluated using genomic DNA samples of *C. albicans* and *C. auris* (10⁷ cells/mL each). (B) Reaction temperature optimization (60–65 °C) for the Candida Pan/auris LAMP assay was performed using the same genomic DNA samples (10⁷ cells/mL each). (C) Performance of the optimized assay was assessed using *C. albicans* genomic DNA (10⁷ cells/mL), *C. auris* genomic DNA (10⁷ cells/mL), and a no-template control (NTC; distilled water).

### Comparison of the detection limits of the *Candida* Pan/*auris* LAMP assay and conventional PCR

The clinically relevant detection performance of the Candida Pan/auris LAMP assay was evaluated using (i) quantified genomic DNA extracted from culture-derived *Candida* cells and serially diluted to defined DNA inputs, and (ii) serially diluted *Candida* cell suspensions spiked into contrived specimens consisting of urine, swab, and whole-blood matrices, followed by nucleic acid extraction. The results were compared with Candida Pan qPCR and *C. auris* qPCR (Tables 2 and 3, S1 Fig). For *C. albicans*, qPCR produced detectable amplification at approximately 10⁴ cells/mL, whereas the Candida Pan LAMP probe produced detectable signals at approximately 10⁵ cells/mL. The *C. auris*-specific probe showed no amplification for any *C. albicans* samples, confirming complete analytical specificity. For *C. auris*, qPCR detected amplification at approximately 10³ cells/mL across all specimen types. In contrast, the *C. auris*-specific LAMP probe consistently detected *C. auris* at approximately 10²–10³ cells/mL in both culture-derived DNA and spiked urine, swab, and blood samples. The Candida Pan probe detected *C. auris* at approximately 10⁴–10⁵ cells/mL, consistent with its broad detection range. Although the *C. auris*-specific LAMP probe showed detection at one dilution lower in urine and blood samples, this minor difference is most likely attributable to variation in nucleic acid recovery during the extraction process at low cell concentrations. Overall, the LAMP assay demonstrated analytical performance comparable to that of qPCR across specimen types.

**Table 2:** Analytical performance evaluation of the *Candida* Pan qPCR and the *Candida* Pan/*auris* LAMP assay for cultured *C. albicans DNA and C. albicans-*spiked contrived specimens.

*Candida albicans*	PCR analysis	Primer sets	Total Concentration [Cells/mL (pg/reaction)]								
			10^7^ ^(16000)^	10^6^ ^(1600)^	10^5^ ^(160)^	10^4^ ^(16)^	10^3^ ^(1.6)^	10^2^ ^(0.16)^	10^1^ ^(0.016)^	10^0^ ^*(0.0016)*^	DW
Culture samples	qPCR (Ct)	*Candida* Pan (FAM)	20.88 ±0.16	23.40 ±0.14	26.17 ±0.08	28.66 ±1.82	ND	ND	ND	ND	ND
	*Candida* Pan/*auris* LAMP (Tt)	*Candida* Pan (FAM)	14.34 ±0.27	14.49 ±0.28	18.76 ±3.09	ND	ND	ND	ND	ND	ND
		*C. auris* (Cy5)	ND	ND	ND	ND	ND	ND	ND	ND	ND
Urine samples	qPCR (Ct)	*Candida* Pan (FAM)	22.36 ±0.51	23.18 ±0.15	27.56 ±0.16	32.84 ±0.79	ND	ND	ND	ND	ND
	*Candida* Pan/*auris* LAMP (Tt)	*Candida* Pan (FAM)	14.31 ±0.08	15.64 ±0.71	19.36 ±0.38	ND	ND	ND	ND	ND	ND
		*C. auris* (Cy5)	ND	ND	ND	ND	ND	ND	ND	ND	ND
Swab samples	qPCR (Ct)	*Candida* Pan (FAM)	19.61 ±0.08	22.81 ±0.38	26.48 ±0.37	30.79 ±1.54	ND	ND	ND	ND	ND
	*Candida* Pan/*auris* LAMP (Tt)	*Candida* Pan (FAM)	14.74 ±0.09	17.21 ±1.62	21.09 ±1.76	ND	ND	ND	ND	ND	ND
		*C. auris* (Cy5)	ND	ND	ND	ND	ND	ND	ND	ND	ND
Blood samples	qPCR (Ct)	*Candida* Pan (FAM)	18.72 ±0.07	22.26 ±0.37	26.54 ±1.01	31.15 ±2.00	ND	ND	ND	ND	ND
	*Candida* Pan/*auris* LAMP (Tt)	*Candida* Pan (FAM)	15.88 ±0.44	17.40 ±0.11	27.20 ±8.72	ND	ND	ND	ND	ND	ND
		*C. auris* (Cy5)	ND	ND	ND	ND	ND	ND	ND	ND	ND

Ct, cycle threshold (qPCR); Tt, threshold time in minute (LAMP); ND, not detected; Values are presented as mean ± SD (n = 3).

Threshold was determined based on the baseline fluorescence of the negative control

**Table 3:** Analytical performance evaluation of the *C. auris* qPCR and the *Candida* Pan/*auris* LAMP assay for cultured *C. auris DNA and C. auris-*spiked contrived specimens.

*Candida auris*	PCR analysis	Primer sets	Total Concentration [Cells/mL (pg/reaction)]								
			10^7^ ^(16000)^	10^6^ ^(1600)^	10^5^ ^(160)^	10^4^ ^(16)^	10^3^ ^(1.6)^	10^2^ ^(0.16)^	10^1^ ^(0.016)^	10^0^ ^(0.0016)^	DW
Culture samples	qPCR (Ct)	*C. auris (FAM)*	16.15 ±1.04	19.66 ±0.68	23.16 ±0.65	27.16 ±0.69	31.52 ±0.68	ND	ND	ND	ND
	*Candida* Pan/*auris* LAMP (Tt)	*Candida* Pan (FAM)	11.29 ±0.02	11.92 ±0.03	13.84 ±0.65	16.85 ±3.88	ND	ND	ND	ND	ND
		*C. auris* (Cy5)	10.32 ±0.01	10.69 ±0.12	11.80 ±0.11	13.30 ±0.10	17.23 ±0.81	ND	ND	ND	ND
Urine samples	qPCR (Ct)	*C. auris (FAM)*	15.06 ±0.33	18.34 ±0.56	25.03 ±5.45	25.32 ±0.04	29.48 ±0.49	ND	ND	ND	ND
	*Candida* Pan/*auris* LAMP (Tt)	*Candida* Pan (FAM)	13.46 ±0.35	14.65 ±0.50	15.99 ±0.22	16.94 ±0.78	ND	ND	ND	ND	ND
		*C. auris* (Cy5)	9.09 ±0.02	10.13 ±0.05	11.31 ±0.17	12.50 ±0.07	13.96 ±0.18	16.16 ±0.11	ND	ND	ND
*Swab samples*	qPCR (Ct)	*C. auris (FAM)*	17.10 ±0.04	20.47 ±0.11	23.86 ±0.37	28.27 ±0.21	32.56 ±1.33	ND	ND	ND	ND
	*Candida* Pan/*auris* LAMP (Tt)	*Candida* Pan (FAM)	14.49 ±0.15	15.10 ±0.15	16.53 ±0.91	18.40 ±2.95	ND	ND	ND	ND	ND
		*C. auris* (Cy5)	9.47 ±0.02	10.28 ±0.04	11.45 ±0.16	12.88 ±0.14	14.54 ±0.66	ND	ND	ND	ND
*Blood samples*	qPCR (Ct)	*C. auris (FAM)*	18.79 ±1.42	22.14 ±2.07	23.71 ±0.40	27.16 ±0.15	29.89 ±0.69	ND	ND	ND	ND
	*Candida* Pan/*auris* LAMP (Tt)	*Candida* Pan (FAM)	13.56 ±0.19	14.27 ±0.34	15.82 ±0.31	17.09 ±1.13	21.83 ±5.31	ND	ND	ND	ND
		*C. auris* (Cy5)	10.02 ±0.19	10.57 ±0.15	11.72 ±0.19	13.13 ±0.22	14.16 ±0.20	16.40 ±1.12	ND	ND	ND

Ct, cycle threshold (qPCR); Tt, threshold time in minute (LAMP); ND, not detected; Values are presented as mean ± SD (n = 3).

Threshold was determined based on the baseline fluorescence of the negative control

### Sensitivity and Specificity of the *Candida* Pan/*auris* LAMP Assay and Pan qPCR in *Candida*-positive and -negative Clinical Specimens

The clinical performance of the multiplex *Candida* Pan/*auris* LAMP assay was evaluated using *Candida*-positive clinical specimens (n = 35; whole blood, n = 9; urine, n = 13; ear swabs, n = 13) and negative control specimens (n = 94; urine, n = 30; swab, n = 32; blood, n = 32) (Table 4). For *Candida* spp. detection, the *Candida* Pan probe of the LAMP assay showed a clinical sensitivity of 97.14% (34/35), compared with 91.14% (32/35) for the *Candida* pan qPCR assay. At the species level, both assays correctly identified *C. albicans* (n = 6), *C. glabrata* (n = 6), *C. tropicalis* (n = 7), *C. parapsilosis* (n = 5), and *C. krusei* (n = 1). However, *C. utilis* (n = 1) was detected only by the *Candida* Pan qPCR. Among *C. auris*–positive specimens (n = 9), the Pan/*auris* LAMP assay detected all cases (9/9; 100%), while the *Candida* Pan qPCR detected 6/9 (66.7%), demonstrating superior clinical sensitivity of the LAMP assay for *C. auris*. The *C. auris*–specific Cy5 probe generated positive signals exclusively for *C. auris*, with no cross-reactivity among non-*auris Candida* species. All negative control specimens (n = 94) tested negative in both assays, confirming 100% clinical specificity.

**Table 4:** Sensitivity and specificity of the *Candida* Pan/*auris* LAMP assay and *Candida* pan qPCR using clinical specimens.

Clinical specimens		*Candida* Pan qPCR	*Candida* Pan/*auris* LAMP assay	
		*Candida* Pan (FAM)	*Candida* Pan (FAM)	*Candida auris* (Cy5)
*Candida Spp.* (n=35)	TP/FN	32/3	34/1	9/26
	Sensitivity	91.14%	97.14%	100%
	Specificity	-	-	100%
*C. albicans* (n=6)	TP/FN	6/0	6/0	0/6
	Sensitivity	100%	100%	-
	Specificity	-	-	100%
*C. glabrata* (n=6)	TP/FN	6/0	6/0	0/6
	Sensitivity	100%	100%	-
	Specificity	-	-	100%
*C. tropicalis* (n=7)	TP/FN	7/0	7/0	0/7
	Sensitivity	100%	100%	-
	Specificity	-	-	100%
*C. auris* (n=9)	TP/FN	6/3	9/0	9/0
	Sensitivity	66.66%	100%	100%
	Specificity	-	-	
*C. parapsilosis* (n=5)	TP/FN	5/0	5/0	5/0
	Sensitivity	100%	100%	-
	Specificity	-	-	100%
*C. krusei* (n=1)	TP/FN	1/0	1/0	0/1
	Sensitivity	100%	100%	-
	Specificity	-	-	100%
*C. utilis* (n=1)	TP/FN	1/0	0/1	0/1
	Sensitivity	100%	0%	-
	Specificity	-	-	100%
Negative Negative control specimens (n=94)	TP/FN	0/100	0/100	0/100
	Sensitivity	-	-	-
	Specificity	100%	100%	100%

LAMP: Loop-mediated isothermal amplification; TP: true positive; FN: false negative

### Cross-reactivity test

To evaluate potential cross-reactivity, the Candida Pan/auris LAMP assay was tested against non-*Candida* bacterial isolates, including *Escherichia coli, Enterococcus faecium, Klebsiella spp., Staphylococcus aureus, and Staphylococcus epidermidis* (Table 5). No amplification was observed with any of these bacterial samples, demonstrating that the assay specifically detects Candida species without cross-reactivity. Notably, 12–32 ng of non-target bacterial DNA per reaction did not produce any detectable amplification signal, further supporting the high analytical specificity of the assay.

**Table 5:** Cross-reactivity of the *Candida* Pan/IC LAMP assay against other bacterial infection samples.

		*Candida* Pan/*auris* LAMP	
		*Candida* Pan (FAM)	*Candida auris* (Cy5)
Samples	DNA Concentration (ng/reaction)	Tt	Tt
*Escherichia coli*	28	ND	ND
*Enterococcus faecium*	20	ND	ND
*E. faecalis*	12	ND	ND
*Staphylococcus aureus*	18	ND	ND
*Staphylococcus epidermidis*	20	ND	ND
*Streptococcus pneumoniae*	14	ND	ND
*Pseudomonas aeruginosa*	32	ND	ND

Tt: Threshold time in minute; ND, not detected

Threshold was determined based on the baseline fluorescence of the negative control

## Discussion

*C. auris* has emerged as a major global public health threat due to its environmental persistence, efficient human-to-human transmission, and multidrug resistance. Accordingly, there is a growing need for diagnostic tools that are both rapid and highly sensitive, particularly for active surveillance and outbreak control [18–21]. Recent guidance-oriented reviews have further emphasized that rapid laboratory identification and colonization screening are central to infection prevention and control, because delayed recognition may facilitate nosocomial transmission and environmental persistence [22]. In a comprehensive review of studies published between 2020 and 2024, Wong et al. reported that real-time PCR assays, including both commercial kits and laboratory-developed tests, exhibit sensitivities ranging from 44% to 100% and specificities from 92% to 100% [18]. These findings highlight the substantial heterogeneity in PCR-based diagnostic performance and underscore the need for alternative or complementary approaches, particularly for active surveillance and early detection. This variability is also supported by recent comparative data showing meaningful differences in analytical sensitivity, specificity, and cross-reactivity even among currently available real-time PCR assays for *C. auris* detection [23].

Within this broader diagnostic landscape, LAMP-based assays for *C. auris* detection have also been introduced into clinical practice. Hernández Felices et al. evaluated the Eazyplex® *C. auris* LAMP assay for direct detection of colonized patients and reported high specificity and good sensitivity in surveillance swabs, with turnaround times suitable for near-patient testing [21]. While this commercial platform simplifies workflow by integrating extraction and amplification into a cartridge-based system, it is designed to target *C. auris* alone and does not provide broad-range *Candida* screening capability. Similarly, Yamamoto *et al*. recently evaluated the LAMPAuris assay and demonstrated rapid and specific detection of *C. auris* across multiple clades and in clinical skin-swab specimens, supporting the feasibility of single-target isothermal amplification for surveillance-oriented testing. While these previously reported LAMP assays provide practical advantages for rapid detection, they were generally designed as single-target assays intended solely for species-specific identification of *C. auri*s and do not provide broad-range *Candida* screening capability [24].

Previous LAMP-based assays for *C. auris* detection have generally been developed as single-target assays intended solely for species-specific identification of *C. auris*. Earlier studies [25] demonstrated that such assays can provide rapid and specific detection, particularly in surveillance swab specimens, supporting their potential utility for infection control screening. However, their diagnostic scope remains limited because they do not simultaneously provide information on the broader presence of *Candida* species in a specimen. In contrast, the *Candida* Pan/auris LAMP assay developed in the present study was specifically designed as a dual-target multiplex assay that enables both broad-range *Candida* screening and species-specific identification of *C. auris* within a single reaction.

In this context, the Candida Pan/auris LAMP assay developed in the present study was designed to enable simultaneous broad-range Candida screening and species-specific identification of *C. auris* within a single reaction. Because multiplex amplification can be affected by reduced amplification efficiency resulting from primer competition, we systematically optimized the design of the *C. auris*–specific LAMP primers and probe, together with the reaction conditions. Through this optimization process, a condition using a 1:1 ratio of the Pan and *C. auris*–specific primer sets with incubation at 64 °C was identified as optimal. Under this condition, *C. auris* was consistently detected at approximately 10²–10³ cells/mL in culture-derived DNA and in spiked urine, swab, and blood matrices, even in the multiplex reaction format. This represents a 1–2 log improvement in analytical sensitivity compared with the *C. auris*–specific qPCR assay, which exhibited a detection limit of 10⁴–10⁵ cells/mL. With stable detection performance confirmed under multiplex conditions, the assay provides a structural basis for performing distinct diagnostic functions within a single reaction. Specifically, the Candida Pan probe can be used to screen for the presence of Candida DNA in clinical specimens, while the *C. auris*–specific probe enables definitive species-level identification of this high-priority pathogen within the same reaction. Accordingly, this dual-target design overcomes key limitations of previously reported single-target *C. auris* molecular assays, which are unable to simultaneously assess overall Candida presence and *C. auris* status [18].

In an evaluation of 35 *Candida*-positive clinical specimens, the Pan LAMP probe showed slightly higher overall Candida detection (97.14%) than Pan qPCR (91.14%). More importantly, in *C. auris*–positive samples, the multiplex LAMP assay detected all nine cases (100%), whereas the corresponding qPCR detected six of nine (66.7%). These findings suggest that the LAMP-based approach may provide more consistent detection of low-level C. auris colonization than qPCR. In addition, the assay showed no cross-reactivity with the other clinically relevant *Candida* species or with a panel of common bacterial co-pathogens, including *Staphylococcus aureus, Staphylococcus epidermidis, Enterococcus faecium, Klebsiella species*, and *Escherichia coli*. Taken together, these results support reliable interpretation of results in infection surveillance settings. From an infection prevention and control perspective, diagnostic accuracy for *C. auris* is a critical factor directly linked to containment of nosocomial transmission. In particular, false‐negative *C. auris* results may fail to identify colonized or early-infection patients, allowing them to remain in general wards and thereby increasing the risk of ongoing hospital transmission [20,26]. Conversely, false-positive results are also clinically relevant, as they may lead to unnecessary patient isolation and inefficient use of healthcare resources. Taken together, the ability of this LAMP assay to detect low-level colonization more consistently than qPCR supports its potential utility as a primary screening tool in active surveillance programs for high-risk units. Although further studies involving larger clinical cohorts are warranted, the analytical and clinical performance observed in this study suggests the potential role of this assay in *C. auris* infection control strategies.

From a practical standpoint, the workflow of the *Candida* Pan/auris LAMP assay aligns well with operational needs in infection-control programs. Many institutions in endemic or outbreak settings have adopted routine skin, nares, or groin surveillance using culture plus qPCR-based methods, which can be costly and labor-intensive and usually require batched processing to remain cost-effective [19,27]. Our data indicate that a LAMP-based strategy could reduce equipment costs by eliminating the need for real-time thermocyclers and may permit smaller, more frequent batches without substantially increasing overall expense. In addition, the ability to detect low-level colonization across multiple specimen types suggests that the assay could be deployed flexibly for admission screening, point-prevalence surveys, and targeted surveillance of high-risk contacts.

This study had several limitations. First, the number of *C. auris*–positive clinical specimens included in this evaluation was limited (n = 9). These clinical specimens corresponded to all cases from which *C. auris* clinical isolates were recovered at our institution during the study period and reflect the low local prevalence rather than any deliberate restriction of sample size. Although the observed 100% sensitivity in this cohort is encouraging, the number of positive specimens was small. In addition, the *Candida* Pan/*auris* LAMP assay showed a lower limit of detection than the comparator real-time PCR assay in this study. However, larger multi-center evaluations across both high- and low-prevalence regions and diverse clades are needed to confirm the robustness and generalizability of our findings and to determine whether this apparent analytical advantage is maintained in broader clinical settings. Second, although LAMP is inherently simpler and more efficient than conventional PCR, our evaluation was performed using fluorescence instruments typically used for PCR, and visual or naked-eye readout formats were not assessed. As a result, the full operational advantages of LAMP were not realized. Future studies using portable, low-cost LAMP platforms or ready-to-use kits may better demonstrate its simplicity, rapid turnaround, and cost-effectiveness, particularly for deployment in Tier-2 or Tier-3 laboratory settings. Third, we did not evaluate assay performance using environmental samples (e.g., high-touch surfaces, reusable equipment), which are increasingly recognized as important reservoirs for *C. auris* transmission in healthcare settings. In light of the organism’s marked environmental resilience, future work should investigate the applicability of this assay to environmental surveillance in healthcare facilities.

In conclusion, the multiplex *Candida* Pan/*auris* LAMP assay developed in this study provides a rapid, highly sensitive, and cost-effective alternative to existing diagnostic modalities for *C. auris*. By combining broad-range *Candida* detection with highly sensitive, species-specific *C. auris* identification in a single isothermal reaction, and by demonstrating improved analytical and clinical sensitivity compared with qPCR, this assay is well positioned to support timely isolation, cohorting strategies, and antifungal stewardship. As LAMP-based *C. auris* diagnostics continue to evolve—including both commercial platforms and laboratory-developed tests—our findings further support the emerging evidence that isothermal amplification can play a central role in front-line screening and control of this multidrug-resistant fungal pathogen.

## Supporting information

S1 Fig Representative amplification curves for analytical sensitivity (LOD) determination of the *Candida* Pan/*auris* assay. Representative amplification curves demonstrating the analytical sensitivity of the *Candida* Pan*/auris* assay for *C. albicans* (A) and *C. auris* (B). For cultured samples, genomic DNA was first extracted from 1.0 × 10⁷ cells, quantified, and subsequently serially diluted to generate defined DNA inputs prior to amplification. For contrived specimens consisting of urine, swab, and whole-blood matrices, serially diluted *Candida* cell suspensions (10⁷–10⁰ cells/mL) were prepared, spiked into each matrix, and subjected to individual nucleic acid extraction prior to amplification. For each specimen type, qPCR amplification curves are shown in the upper panels and LAMP amplification curves in the lower panels. In the LAMP assay, green curves represent the *Candida* Pan probe and purple curves represent the *C. auris*-specific probe. Dilution levels are indicated adjacent to representative curves. Curves shown represent one of three independent experiments (n = 3). The limit of detection (LOD) was defined as the lowest concentration consistently detected across replicates, as summarized in Tables 2 and 3.
(TIF)
