# Supplementary figures and images for: Development of a Loop-Mediated Isothermal Amplification (LAMP) for the screening of Candida auris
Source: PLoS One. 2026 Apr 24;21(4):e0348003. doi: 10.1371/journal.pone.0348003 (PMC13108731; doi:10.1371/journal.pone.0348003)

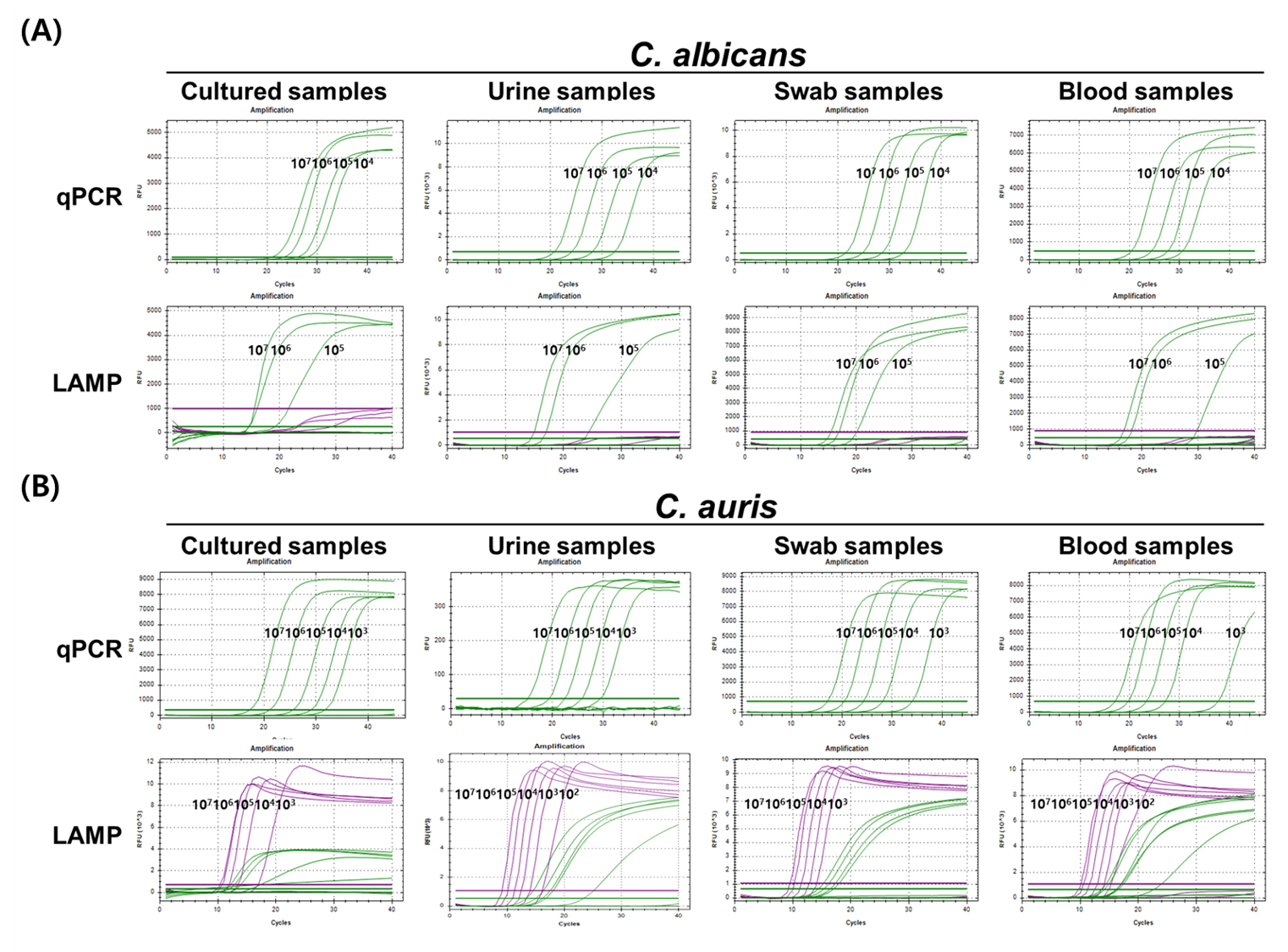

Supplement: S1 Fig — Representative amplification curves demonstrating the analytical sensitivity of the Candida Pan/auris assay for C. albicans (A) and C. auris (B). For cultured samples, genomic DNA was first extracted from 1.0 × 10⁷ cells, quantified, and subsequently serially diluted to generate defined DNA inputs prior to amplification. For contrived specimens consisting of urine, swab, and whole-blood matrices, serially diluted Candida cell suspensions (10⁷–10⁰ cells/mL) were prepared, spiked into each matrix, and subjected to individual nucleic acid extraction prior to amplification. For each specimen type, qPCR amplification curves are shown in the upper panels and LAMP amplification curves in the lower panels. In the LAMP assay, green curves represent the Candida Pan probe and purple curves represent the C. auris-specific probe. Dilution levels are indicated adjacent to representative curves. Curves shown represent one of three independent experiments (n = 3). The limit of detection (LOD) was defined as the lowest concentration consistently detected across replicates, as summarized in Tables 2 and 3. (TIF) [file pone.0348003.s001.tif]
